# Effect of whole body vibration therapy on circulating serotonin levels in an ovariectomized rat model of osteoporosis

**Published:** 2014-01

**Authors:** Qiu-Shi Wei, Li Huang, Xian-Hong Chen, Hai-Bin Wang, Wei-Shan Sun, Shao-Chuan Huo., Zi-Qi Li, Wei-Min Deng

**Affiliations:** 1 Department of Rehabilitation, General Hospital of Guangzhou Military Command of PLA, Guangzhou, China; 2 Key Laboratory of Orthopaedics & Traumatology, Guangzhou University of Traditional Chinese Medicine, Guangzhou, China

**Keywords:** Osteoporosis, Ovariectomy, Serotonin, Whole body vibration

## Abstract

***Objective(s):*** Studies have reported that whole body vibration (WBV) played a vital role in bone remodeling. Circulating serotonin is also involved in negative regulating bone mass in rodents and humans. However, both WBV and inhibition of serotonin biosynthesis may suppress receptor activator of nuclear factor-kappaB ligand (RANKL)-induced osteoclastogenesis *in vitro*. The purpose of the current study was to investigate the effect of WBV therapy on the levels of serum serotonin in ovariectomized rats.

***Materials and Methods:*** Thirty-six-month-old female Sprague Dawley rats weighing 276.15±37.75 g were ovariectomized to induce osteoporosis, and another ten rats underwent sham operation to establish sham control (SHAM) group. After 3 months, ovariectomized rats were divided into three subgroups and then separately treated with WBV, Alendronate (ALN) and normal saline (OVX), SHAM group was given normal saline. After 6 weeks of treatment, rats were sacrificed. Serum serotonin, RANKL, bone turnover markers, and bone mineral density (BMD), bone strength were evaluated.

***Results:*** The serum serotonin level was significantly lower in WBV group than OVX and ALN groups (*P*<0.05 and *P*<0.001). RANKL levels significantly decreased in WBV and ALN groups compared to OVX group (*P*<0.001 for both). BMD and biomechanical parameters of femur significantly increased (*P*<0.05 for both) and bone turnover levels decreased (*P*<0.001 for both) in WBV group compared to OVX group.

***Conclusion:*** These data indicated that WBV enhanced the bone strength and BMD in ovariectomized rats most likely by reducing the levels of circulating serotonin.

## Introduction

Postmenopausal osteoporosis (PMO) is a syste-mic skeletal impairment characterized by a decrease in bone mass, bone strength, and disruption of bone microarchitecture, leading to increase bone fragility and the propensity of fracture, which is associated with an increase in bone resorption outpacing bone formation induced by estrogen deficiency (1, 2). High bone resorption is caused by enhancing osteoclastic activity. Therefore, suppressing osteoclastogenesis is the main treatment principle for PMO.

Studies have shown gut-derived serotonin (GDS) is an important regulatory factor of inhibiting osteo-blast proliferation and bone formation (3, 4), enhancing osteoclast differentiation and bone resorption *in vivo* as well as *in vitro* (5). These findings are consistent with recent studies showing that higher levels of circulating serotonin may increase bone turnover and reduce bone formation in humans (6). Therefore, GDS has an important local role in bone, as inhibition of GDS synthesis can reduce bone turnover levels and block osteoclast differentiation.

The great anabolic potential of mechanical loading, as a natural factor, that plays a key role in maintaining bone morphology and strength in both human and animal skeletons has long been recog-nized (7, 8). Low-magnitude high-frequency loading via whole body vibration (WBV), as a novel and non-invasive oscillatory stimulation, has been displayed an enhancement of bone strength and bone mass in ovariectomized rats (8, 9). However, its effect on bone formation *in vivo *and underlying mechanism remains unclear. Recent studies have reported that WBV has influence on osteoclast differentiation *in vitro* and restrains osteoclastogenesis (10, 11). Ideally, WBV exhibits non-pharmacologically inhibit-ory effects on osteoclasts, so it is necessary to explore the evidence- and mechanistic based study to facilitate the use of WBV in the prevention and treatment of postmenopausal osteoporosis. In the present study, based on the understanding of GDS, we hypothesized that WBV could stimulate bone formation in ovariectomized rats both by both down-regulating the expression of peripheral serotonin and blocking RANKL-induced osteoclast differentiation.

Our objective was to explore the effect of WBV on the level of serotonin in the blood during bone remodeling process in an estrogen deficient model of osteoporosis. We hypothesized that WBV may inhibit circulating serotonin biosynthesis and promote bone anabolism, which in turn could mitigate bone deterioration under estrogen deficient condition. To test this hypothesis, we established the ovariectom-ized model. WBV was applied to rats for a treatment period of days. Meanwhile, alendronate, the first-line anti-resorptive drug for PMO, was compared to observe effectiveness (12). We then evaluated the bone anabolism by analyzing the BMD, bone strength, the levels of serum serotonin, RANKL, and bone turnover markers.

## Materials and Methods

***Animals***

A homogeneous group of 40 female Sprague Dawley rats (6-month-old) were purchased from Guangzhou University of Traditional Chinese Medicine Laboratory Animal Center (SCXK-Guangdong-2008-0020). The rats were housed in standard cages at room temperature and acclimatized for one week with a normal 12 hr light-dark cycle and free access to water and normal rat chow diet. Humane care was performed according to the Guide for the Care and Use of Laboratory Animals, published by the US National Institutes of Health (13). The rat ovariectomy experiment was approved by the Ethical Committee of General Hospital of Guangzhou Military Command of PLA.

***Experimental design***

After one week of acclimatization, the rats were randomly divided into two groups in which rats were bilaterally ovariectomized (OVX, 30 rats) and sham operated (SHAM, 10 rats) at the age of 6 months. Surgery was performed through dorsal approach under general anesthesia with intraperitoneal 10% chloral hydrate injection (3.3 ml/kg). After surgery, rats were untreated for three months.

Three months following the surgery, the OVX rats developed osteoporosis according to previous studies (14). OVX groups (n=30) were treated with whole body vibration (WBV, n=10) or Alendronate (ALN, n=10) or normal saline (OVX, n=10), and SHAM group (SHAM, n=10) was given normal saline. The following was the introduction of WBV: A experimental vibration platform (Jnvent 1000, Jnvent Medical, Inc. Somerset, USA) was performed two times a day, 20 min with 5 min rest at mid-point (10 min on − 5 min off − 10 min on), 5 days per week, a total of 6 weeks (15). A plastic cage was fixed on the periphery of vibration platform which had the capacity to hold three rats. Rats could move freely in the cage during vibration. The device worked at a frequency of 30−35 Hz and an acceleration of 0.3 g (16-18), which was confirmed by Guangdong Medical Devices Quality Surveillance and Test Institute (NO:JK083007).

The alendronate was administrated 7mg/kg per week orally according to Chen *et al* studies (19). WBV and ALN were started 3 month after surgery and lasted for a 6-week period. In addition, rats' body weights were monitored using a JJ500 electronic balance (**STIFCC, **Changshou, china) every week to adjust the administrated dose.

At the end of experimental, rats were anesthetized with intraperitoneal 10% chloral hydrate injection (3.3 ml/kg) until unconscious and blood samples were taken from abdominal aortic and centrifuged for 15 min at 1,000 x g to obtain serum and stored at -80°C until used for following assays. Left femur samples were wrapped with warm saline gauze and stored at −20°C for bone mineral density (BMD) determination and biomechanical testing.

***Serum*** ***analysis***

The levels of serum serotonin, RANKL, the anabolic marker Procollagen I N-terminal peptide (PINP), and bone resorption marker C-telopeptide of collagen (CTX) were measured by enzyme-linked immunosorbent assay (ELISA) Kit (CUSABIO BIOTECH, Wuhan, china). The sensitivity of serotonin was 0.4 ng/ml, and the intra- and interassay coefficients of variation (CV) were <15%. The sensitivity of RANKL, PINP and CTX kit were 0.4 ng/ml, 15.63 pg/ml, 15.6 pg/ml and 3.9 pg/ml, respectively. There were same intraassay (<8%) and interassay (<15%) precision.

***Bone mineral density examination***

BMD was measured using small-animal special Dual Energy X-ray Absorptiometry (DEXA, Hologic, Inc. USA) with the hand-regional high resolution. The femur was divided into three interested regions including proximal femur, femoral shaft, and distal femur. Proximal femur was defined as a site just proximal to the point where located in the lower edge of the lesser trochanter. Femoral shaft was defined as a 2 cm long site just proximal to distal around the femoral shaft midpoint. Distal femur was defined as a site just distal to the junction between femoral condyle cancellous bone and femoral diaphysis cortical bone. After measuring BMD, the femur was stored at −20°C for subsequent biomechanical testing.

**Table 1:** Basic characteristics of experimental and control groups

	SHAM+Saline	OVX+Saline		OVX+WBV		OVX+ALN
Body weight (g)						
At the beginning of study	278.35±45.25	276.81±34.29	270.40±27.92		279.05±29.71	
3 months after surgery	333.53±34.81 *†*	413.24±32.08 *†*	418.73±47.80 *†*		419.95±42.33 *†*	
6 weeks after treatment	360.96±31.02	466.93±25.35 *††*	430.67±42.25		457.28±31.66 *††*	
BMD (g/cm^2^)						
Intact femur	0.280±0.011	0.222±0.016 *	0.252±0.009 *^,#^		0.264±0.013 *^,#,^^△^	
Proximal femur	0.257±0.017	0.218±0.016 *	0.232±0.009 *^,#^		0.239±0.011 *^,#^	
Femoral shaft	0.305±0.012	0.234±0.007 *	0.277±0.010 *^,#^		0.267±0.013 *^,#^	
Distal femur	0.276±0.012	0.229±0.007 *	0.245±0.012 *^,#^		0.256±0.011 *^,#,^^△^	
Biomechanical properties						
Cortical thickness (mm)	0.612±0.034	0.561±0.034 *	0.597±0.038 ^#^		0.607±0.052 ^#^	
Maximal load (N)	123.94±11.88	98.05±13.63 *	117.09±11.22 ^#^		120.97±12.70 ^#^	
Yield load (N)	106.25±16.29	77.46±19.32 *	98.90±21.33 ^#^		102.59±19.65 ^#^	
Stiffness (N/mm)	125.04±14.69	87.56±22.34 *	110.33±23.63 ^#^		115.99±21.51 ^#^	

Data are mean±SD. (Note: *† P*<0.05 *vs *At the beginning of study; *†† P*<0.05 *vs *3 months after surgery; * *P*<0.05 *vs *SHAM+Saline; ^#^ *P*<0.05 *vs *OVX+Saline; ^△^ *P*<0.05 *vs *OVX+WBV.)

BMD = Bone mineral density; OVX = ovariectomized; WBV = whole body vibration; ALN = Alendronate

***Biomechanical testing***

After complete thawing of femoral samples, the femoral diaphysis was tested to failure via three-point bending using a BOSE ElectroForce 3520 biological material testing system (Minnesota, USA) to measure the changes in mechanical properties. A load was applied at the speed of 5 mm/min on the middle of femoral diaphysis on the anterior surface until the bone was fractured (20). A load versus deformation graph was plotted, maximal load and yield load were derived from the gradient of resulting curve.

***Statistical analysis***

The results were presented as mean±SD. The data analysis was performed using SAS version 8.0 (SAS Institute, Cary, NC, USA). One−Way ANOVA and Tukey’s post-hoc test were performed to compare to the results of multiple groups. For the experiments involving two groups, independent-samples T-test was performed. A *P* value of less than 0.05 was considered statistically significant.

## Results

***Body weight***

At the beginning of the experiment, there was no significant difference in body weight among four groups (*P*=0.943), with an average of 276.15 g±37.75 g. 3 months after surgery, the body weight in all groups was considerably increased when compared to the baseline (*P*<0.001 for all groups). However, body weight for OVX and OVX+ treated groups increased significantly (25.1%, *P*<0.001) compared to the SHAM group. After 6 weeks of treatment, SHAM group rats could maintain a steady body weight throughout the study, with only a +8.2% difference from the start of treatment to the end (*P*=0.079). It was significantly lower (−20.1%, *P*<0.001) in SHAM group than other groups, and no differences was observed among OVX and OVX + treated groups. Body weight for OVX and ALN groups at 6 weeks after treatment was significantly higher (*P*=0.001 and *P*=0.038, respectively) than the time of 3 months after surgery, no significant change was found in WBV group (*P*=0.561, Table 1).

***Serum analysis***

Table 2 summarizes the percent difference in serum analysis for each group.

**Figure 1:**
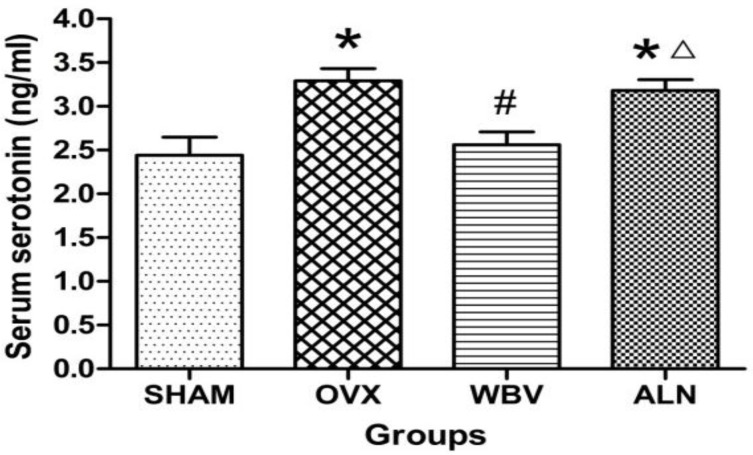
The change differences in serum serotonin levels after 6 weeks of treatment. Serum samples from the rats of the following groups: (1) SHAM + Saline (SHAM), (2) OVX + Saline(OVX), (3) OVX + WBV (WBV), and (4) OVX + ALN (ALN). (n = 10 rats each group). Values are expressed as mean±SD.

Figure 1 shows that the serum concentration of serotonin were significantly lower in SHAM and WBV groups than OVX group (*P*<0.001 for both), and it was identical in SHAM and WBV groups than ALN group (*P*<0.001 for both). There was no significant difference between SHAM and WBV groups (*P*=0.340), and no difference was found between ALN and OVX groups (*P*=0.419).

**Figure 2:**
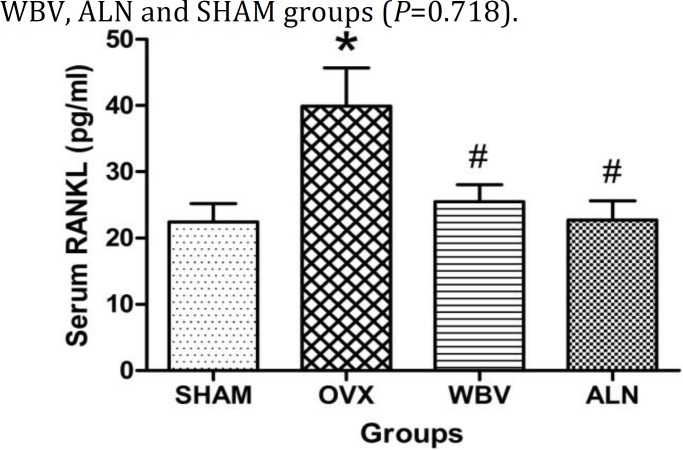
The change differences in serum RANKL levels after 6 weeks of treatment. Serum samples from the rats of the following groups: (1) SHAM + Saline (SHAM), (2) OVX + Saline(OVX), (3) OVX + WBV (WBV), and (4) OVX + ALN (ALN). (n = 10 rats each group). Values are expressed as mean±SD. * *P* < 0.05, versus SHAM group; ^#^ *P* < 0.05, versus OVX group. ^△^ *P* < 0.05, versus WBV group

The levels of RANKL were significantly lower in SHAM, WBV and ALN groups than OVX group (*P*<0.001 for all groups; Figure 2). Although no significant difference was indentified among WBV, ALN, and SHAM groups (*P*=0.715), its levels were still higher in WBV and ALN groups than SHAM group.

**Table 2:** Percentage change in serum serotonin, RANKL and bone turnover markers for each group

	Serotonin	RANKL	P1NP	CTX
OVX+Saline versus SHAM+Saline	34.8% ^###^	77.6% ^###^	106% ^###^	74.4% ^###^
OVX+WBV versus SHAM+Saline	4.9%	4.6%	0.1%	42.7% ^###^
OVX+ALN versus SHAM+Saline	30.3% ^###^	1.2%	－6.1%	40.8% ^###^
OVX+WBV versus OVX+Saline	－22.2% ***	－41.1% ***	－51.4% ***	－18.2% **
OVX+ALN versus OVX+Saline	－3.3%	－43.0% ***	－54.4% ***	－19.3% **
OVX+WBV versus OVX+ALN	－19.5% ^△△△^	3.4%	6.6%	1.3%

Note: ^#^ *P*<0.05, ^##^ *P*<0.01, ^###^ *P*<0.001 *vs *SHAM+Saline; * P<0.05, ** *P*<0.01, *** *P*<0.001 *vs *OVX+Saline; ^△^ *P*<0.05, ^△△^ *P*<0.01, ^△△△^ *P*<0.001 *vs *OVX+ALN.

OVX = ovariectomized; WBV = whole body vibration; ALN = Alendronate

* *P *< 0.05, versus SHAM group; ^#^ *P* < 0.05, versus OVX group. ^△^ *P* < 0.05, versus WBV group

OVX = ovariectomized; WBV = whole body vibration; ALN = Alendronate

**Figure 3:**
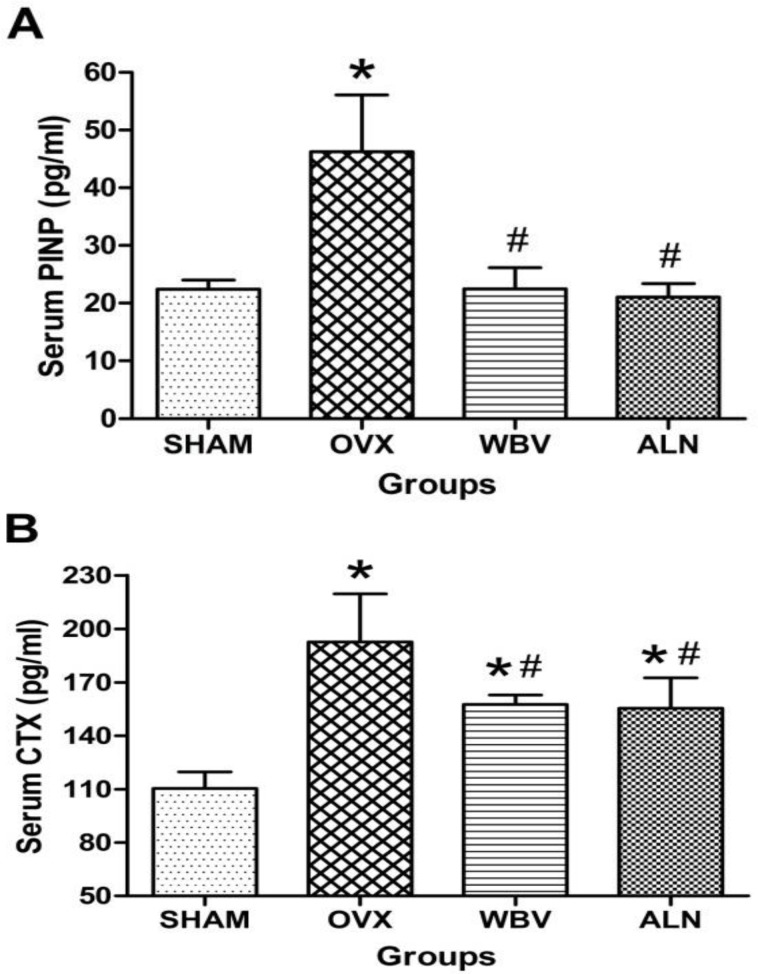
The change differences in bone turnover markers PINP (A) and CTX (B) levels after 6 weeks of treatment. Serum samples from the rats of the following groups: (1) SHAM + Saline (SHAM), (2) OVX + Saline (OVX), (3) OVX + WBV (WBV), and (4) OVX + ALN (ALN). (n = 10 rats each group). Values are expressed as mean ± SD.

Our results showed that serum PINP levels significantly decreased in SHAM, WBV and ALN groups compared to OVX group (*P*<0.001 for all groups; Figure 3A). However, its levels were higher in WBV group and lower in ALN group than SHAM group (*P*=0.982 and *P*=0.147, respectively), although the *p *value was greater than 0.05. Like the PINP level, the CTX levels significantly decreased in SHAM, WBV and ALN groups compared to OVX group (*P*<0.001 for all groups; Figure 3B). However, CTX levels in WBV and ALN groups were higher than SHAM group (*P*<0.001 for both).

***Bone mineral density analysis***

BMD at intact femur and different regions was significantly higher in SHAM, WBV and ALN groups than OVX group (*P*<0.05 for all groups; Table 1). However, their levels were still lower in WBV and ALN groups than SHAM group* (P*<0.05 for both). Compared to ALN group, WBV group had 3.4% (*P*=0.091) increase BMD at femoral shaft, On the contrary, BMD at intact femur, distal femur and proximal femur decreased 4.7%, 4.3% and 3.0%, respectively (*P*<0.05, *P*<0.05, and *P*=0.115, respec-tively).

***Biomechanical properties analysis***

The cortical thickness, maximal load, yield load, and stiffness in femur collected from OVX group were significantly lower than that of SHAM group (−8.1%, −20.9%, −27.1% and −30.0%; *P*<0.001 for all groups). Results are shown in Table 1. Compared to OVX group, WBV and ALN groups significantly increase cortical thickness (6.4% and 8.2%, *P*<0.05 for both), maximal load (19.4% and 23.4%; *P*<0.01 for both), yield load (27.7% and 32.4%; *P*<0.05 for both) and stiffness (26.0% and 32.5%; *P*<0.05 for both). No significant difference was identified among WBV, ALN and SHAM groups (*P*=0.718).

OVX = ovariectomized; WBV = whole body vibration; ALN = Alendronate

## Discussion

In this study, our data demonstrated that whole body vibration (WBV) could partially mitigate adverse changes to bone mass and mechanical properties in ovariectomized rats. Furthermore, WBV caused significant decrease in serum serotonin and RANKL levels compared to OVX group.

* *P* < 0.05, versus SHAM group; ^#^ *P* < 0.05, versus OVX group. ^△^ *P* < 0.05, versus WBV group

OVX = ovariectomized; WBV = whole body vibration; ALN = Alendronate

WBV imitates a natural form of short and repetitive mechanical loading which governs bone strength and bone mineral contents in children as well as maintenance of bone mass in postmeno-pausal women (7, 21). The response of skeletal tissues to mechanical loading depends on factors including magnitude, duration, and rate of the stimulus. Dynamic or cyclic stimuli seem to be more anabolic to static stimuli (15). Because bone cells will accommodate to routine loading, rest periods between cycles of mechanical loading should be inserted to restore bone’s mechanosensitivity (22). At present study, dynamic WBV stimulation has displayed an enhancement of bone strength and BMD in ovariectomized rats. Maximal force, yield force, and stiffness are the most widely used biomechanical parameters for measuring bone strength (23). The BMD at femur was determined using small-animal special DXA. From this, we could obtain total and interested region BMD. Our findings show that biomechanical parameters and BMD of femur had a significant decrease in OVX group versus SHAM group, and these were significantly enhanced after WBV compared to OVX group, although these in WBV group was inferior to SHAM group. However, Sehmisch *et al*, who reported that bone strength and bone mass in ovariectomized rats treated with WBV was identical to those in SHAM group rats (9). It was worth noting that differences could arise due to the targeted skeletal site. Our study addressed the effects of treatment at the femur, while the study of Sehmisch *et al* targeted the vertebral.

Unfortunately, the effect of WBV on bone metabolism *in vivo* and its underlying mechanisms were still not clearly understood. Previous studies had demonstrated WBV could promote bone mesenchymal stromal cells (BMSCs) proliferation and differentiation toward osteogenesis (24). In addition, WBV could also abrogate RANKL-induced osteoclastogenesis (10, 11). RANKL is the key regulator of osteoclast formation and function (25, 26). RANKL expression is upregulated by estrogen deficiency, which suggests that it may play a pivotal role in mediating enhanced bone resorption, bone loss, and bone resorption markers in menopause (27). Both estrogen and RANKL inhibitor modulate osteoclast development by directly blocking RANKL-induced osteoclastogenesis (28, 29). Therefore, the prominent role of RANKL in osteoclastogenesis has made it a potential target in bone diseases charac-terised by excessive bone loss.

Gut-derived serotonin (GDS), the same as peripheral serotonin, is one of the hot issues today (4-6). However, the function of peripheral serotonin in bone has recently been the theme of controversy. Two recent studies have demonstrated that bone phenotypes in TPH1 knockout (KO) mice. TPH1, the rate-limiting enzyme tryptophan hydroxylase 1, was involved in the peripheral serotonin biosynthetic pathway in enterochromaffin cells of the duodenum. Serotonin in circulating blood is nearly absent in TPH1 deficient mice compared to WT mice (30). One shows inhibiting GDS biosynthesis could work to the same degree as an antiresorptive drugs to increase bone mass in ovariectomized rodents through a new anabolic mechanism (31). Another shows no signi-ficant change in bone mass measured by dual-energy X-ray absorptiometry in deficiency peripheral serotonin mice (32). To reassess these conflictive findings, Chabbi-Achengli *et al* distinctively proved that GDS played an important role in osteoclastic differentiation and function *in vivo* as well as *ex vivo*, and inhibition of serotonin decreased bone resor-ption due to fewer osteoclasts in TPH1 KO mice. In addition, osteoclast precursors could express TPH1 and synthesize serotonin in the presence of RANKL, whereas both spleen cells and bone marrow macro-phages from TPH1 KO mice in the presence of RANKL reduced the number of osteoclasts could be rescued by adding serotonin (5). In other words, GDS has an important effect on blocking RANKL-induced osteoclast differentiation *in vivo* and *vitro*. Further-more, it has been reported that the concentration of serotonin in the blood is higher in humans with increased bone turnover and reduced bone forma-tion (6). Therefore, the level of serotonin in the blood may be a marker for diagnosis and treatment of PMO.

, Based on the understanding of GDS and RANKL, the initial hypothesis was confirmed in this study showing that WBV has a positive effect on bone anabolic in ovariectomized rats by directly down-regulating the expression of serum serotonin and RANKL. Our study found that circulating serotonin and RANKL were significantly higher in OVX group than SHAM group. However, RANKL were also significantly lower in WBV group than OVX group. No significant difference was observed between SHAM group and WBV group.

Meanwhile, alendronate was compared to verify the effect of WBV. Anti-resorptive reagents such as alendronate which effectively increase BMD, mean-time, significantly decrease bone turnover markers have been established as the first-line therapy to prevent hip fractures in postmenopausal women with osteoporosis (33). However, recent studies have provided a well-documented association between atypical femoral fractures and long-term oral alendr-onate, so alendronate may not be routinely administered to patients with osteoporosis in clinics (34, 35). Thus, a non-invasive and easy-to-apply intervention is necessary to develop new treatment for osteoporosis. As a non-pharmacological mecha-nical intervention, WBV provides a noninvasive approach to regulate the bone formation, and may be a good alendronate (ALN) replacement therapy. In our experiment, biomechanical properties of rats treated with WBV were equivalent to ALN, intact femur and distal femur BMD in WBV group were weaker than ALN group. However, femoral shaft BMD was higher in WBV group than ALN group, though the *p *value was greater than 0.05. Our study also found that serum serotonin level of rats treated with WBV was significantly lower than with ALN, but no significant difference in serum RANKL levels was observed between WBV and ALN groups. All of these may give us information to understand the under-lying mechanism of WBV to improve osteoporosis by decreasing both the expression of circulating serotonin and RANKL. Moreover, ALN may promote bone formation in ovariectomized rats by directly reducing the expression of serum RANKL. This is in line with Eslami *et al,* who reported that ALN may block human osteoclastogenesis by decreasing RANKL expression in marrow cells (36).

Based on our findings, it is wise to replace ALN with WBV in clinic, although both of them have their strengths and weaknesses in improvement of postmenopausal osteoporosis. However, in order to achieve a better clinical effect, combined use of WBV and ALN might achieve a synergic effect, due to the fact that both of these reagentscan improve osteopo-rosis by increasing BMD. This combination therapy may decrease the dosage of ALN in clinical patients, and probably reduce the side effects with the increased efficacy.

## Conclusion

In summary, the current study indicates that estrogen deficiency results in a significant increase in the expression of circulating serotonin post-ovarie-ctomy. Treatment with WBV reduces the expression of serotonin in the blood of ovariectomized rats. Therefore, WBV, as a non-invasive and non-pharmac-ological therapeutic intervention, effectively prev-ents bone loss in ovariectomized rats by decreasing the expression of circulating serotonin. However, it should be noted that since we did not fully elucidate possible mechanism, further studies need be performed to explore whether circulating serotonin is not merely a phenomena but is really necessary for the therapeutic effect of WBV.
